# P164: Survey on patients’ perception of methicillin-resistant Staphylococcus aureus (MRSA) prevention and control

**DOI:** 10.1186/2047-2994-2-S1-P164

**Published:** 2013-06-20

**Authors:** RFY Chan, SC Fung, SW Chan, KY Chau, MY Yim, MC Li, OT Chau, YK Tze

**Affiliations:** 1 Infection Control Team, United Christian Hospital, The Hospital Authority of Hong Kong, HKSAR, China; 2 Pathology Department, United Christian Hospital, The Hospital Authority of Hong Kong, HKSAR, China

## Introduction

Methicillin-resistant *Staphylococcus aureus* has been endemic in Hong Kong, and accounts for approximately 40-45% of all *Staphylococcus aureus* isolates. A territory-wide admission screening of 7387 patients from Department of Medicine of acute care hospitals in 2011 revealed that 14.2% carried MRSA. Efforts to enhance infection control from all parties, including the patients, are keys to prevention of transmission.

## Objectives

To assess the patients’ level of understanding, prevention and control of MRSA, identify improvement measures from patients’ perspective, and explore the public education in relation to MRSA.

## Methods

A cross-sectional study was conducted in the out-patient clinics of the medical department of an acute regional hospital in Hong Kong in 2012. Face-to-face interview using a structured questionnaire was used.

## Results

A total of 429 patients completed the interview. There were 203 male and 226 female; 65% aged over 40 years old. 253 (59%) have heard of MRSA, mainly from the media (85.8%). Around 50% correctly recognized it as bacteria and 18.2% knew about asymptomatic carriage. 30-40% associated MRSA with pneumonia and soft tissue infection, whereas 40% had no idea of disease association. 63.6% considered antibiotics for treatment and 28.5% had no idea at all. Droplets (37.5%) and direct contact (30.4%) were considered as common routes of MRSA transmission, 39.7% showed no idea. Around 65% perceived that crowded hospital environment and having MRSA patients at neighborhood increase the risk of acquisition. 15-20% regarded surgery, line insertion and total dependency as risk factors. 20% expressed moderate worry about MRSA acquisition. Two-third did not consider visitor and family member's role are extremely important in MRSA control.

## Conclusion

This study showed that patients’ general knowledge on MRSA was low. The main source of information was from the media, and role of patient's visitor or family member in MRSA control is not considered essential. Patients are interested to get further information about MRSA, in particular for transmission and prevention. Enhancement of public knowledge and responsibility on MRSA control via multi-disciplines and multimedia could be helpful in reducing the spread of MRSA.

## Disclosure of interest

None declared

